# Supplementary material for: KRAS regulation by small non-coding RNAs and SNARE proteins
Source: Nat Commun. 2019 Nov 11;10:5118. doi: 10.1038/s41467-019-13106-4 (PMC6848142; doi:10.1038/s41467-019-13106-4)
Supplement: Supplementary file 1 — Supplementary Information [file 41467_2019_13106_MOESM1_ESM.pdf]

KRAS regulation by small non-coding RNAs and SNARE proteins

Authors: Yonglu Che[1,2], Zurab Siprashvili[1,2], Joanna R. Kovalski [1,2], Tiffany Jiang[1], Glenn Wozniak[1], Lara Elcavage[1], Paul A. Khavari* [1,2,3]

Supplementary Information

Supplementary Figure 1

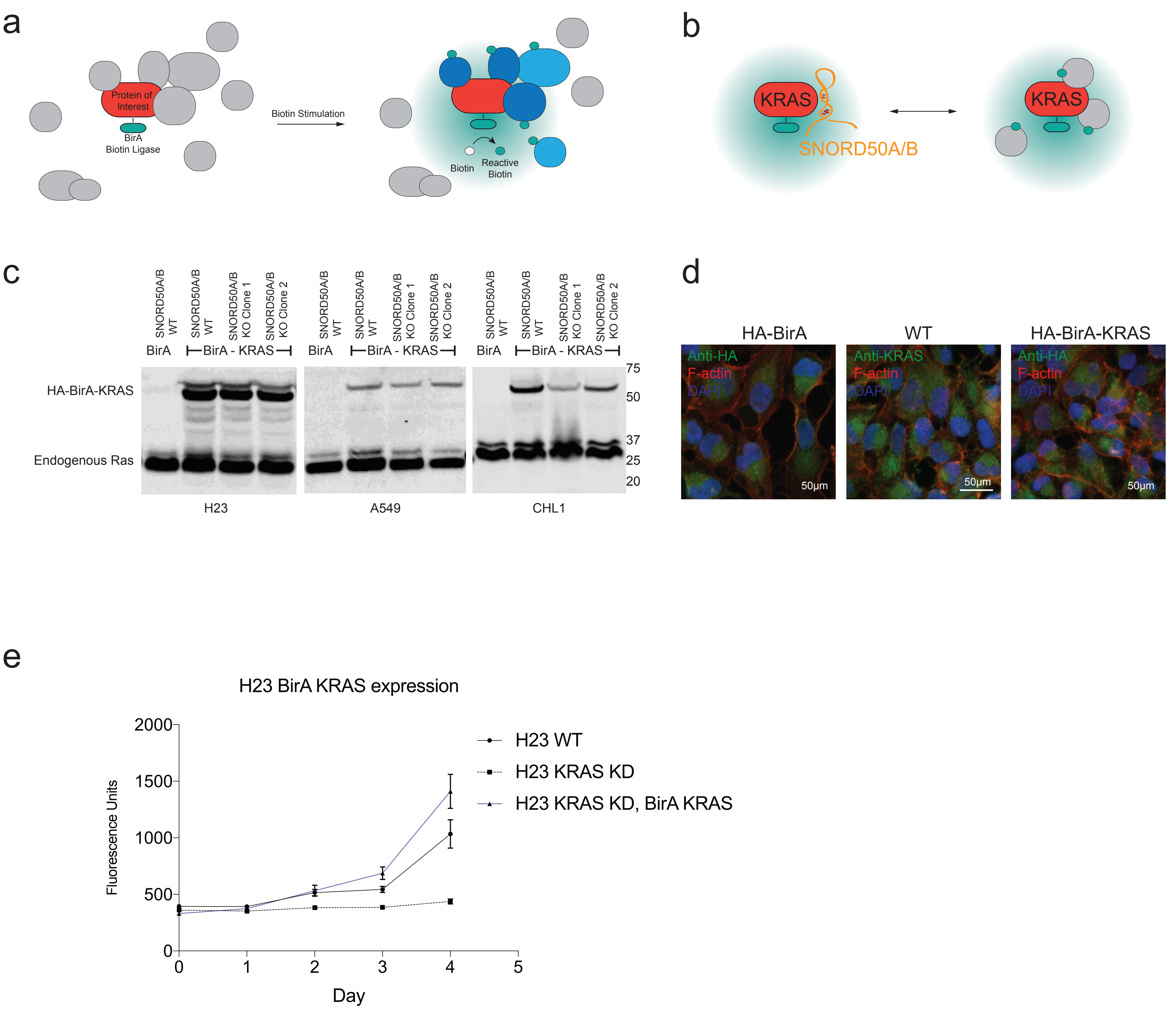

Supplementary Figure 1.

(A) Schematic of proximity protein labeling experiments. (B) Schematic of KRAS proximity proteomics with and without SNORD50A/B. (C) Expression of HA-BirA-KRAS construct relative to endogenous Ras protein levels in H23, A549 and CHL1 cells used for mass spectrometry. (D) Immunofluorescence of HA-BirA, endogenous KRAS, and HA-BirA-KRAS in H23 cells. (E) KRAS KD in WT and HA-BirA-KRAS expressing H23 cells. KRAS KD was performed with a 3'UTR-specific shRNA and WT conditions refer to cells exposed to a scrambled control shRNA. Error bars are s.e.m.

Supplementary Figure 2

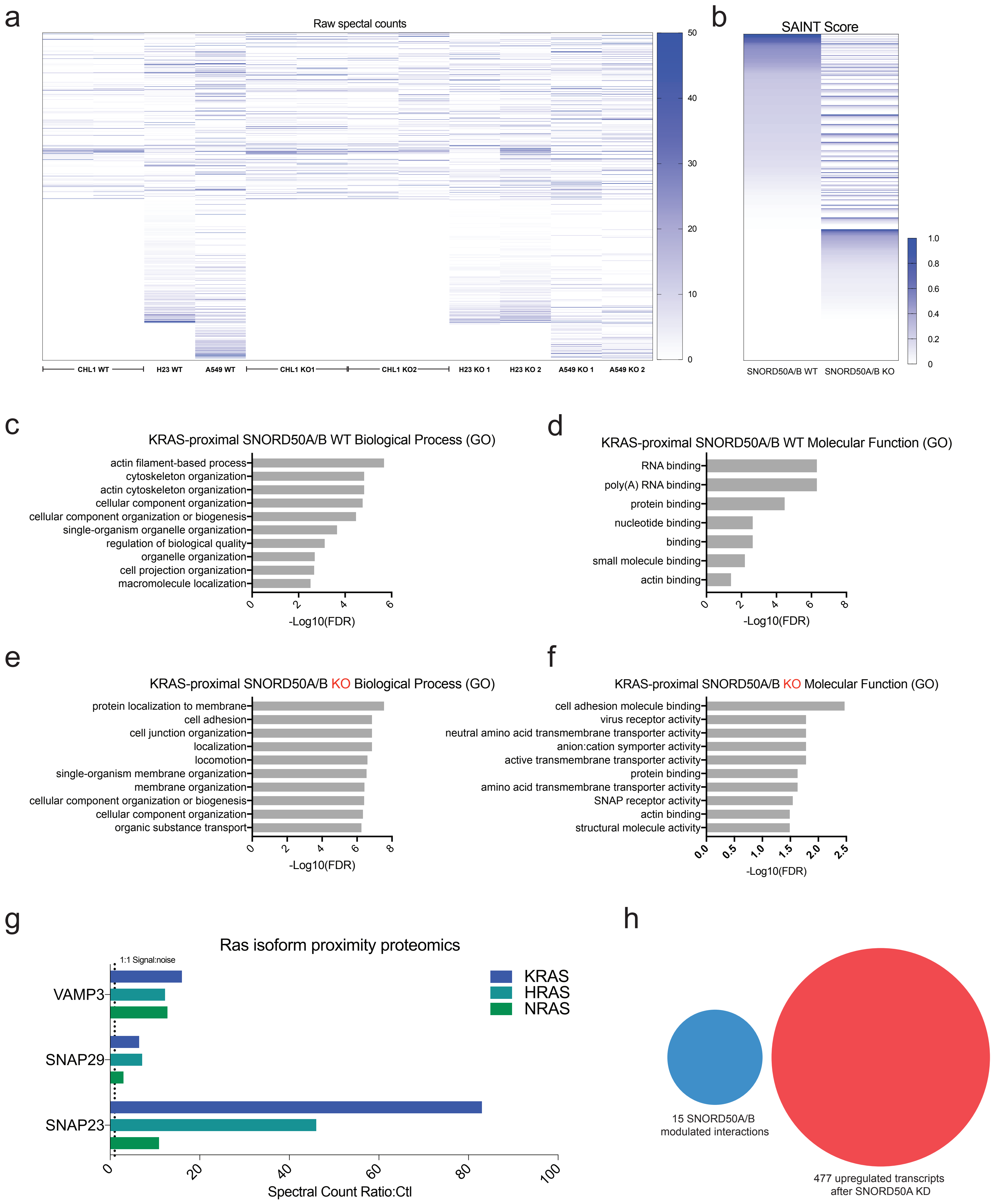

Supplementary Figure 2.
(A) Spectral counts by protein (row) and sample (column) from mass spectrometry analysis of KRAS vicinal proteins. (B) SAINT scores of proteins enriched in proximity of KRAS. (C-F) Gene Ontology (GO) terms enriched in WT and SNORD50A/B KO cell lines. (G) Spectral counts of detect SNAP23, SNAP29, and VAMP3 by mass spectrometry in proximity to HA-BirA-KRAS, HA-BirA-HRAS, and HA-BirA-NRAS. (H) Comparison of increased KRAS proximal interaction in SNORD50A/B KO and transcripts globally found to be increased in expression after SNORD50A KD from Huang et al. 2017[66].

Supplementary Figure 3

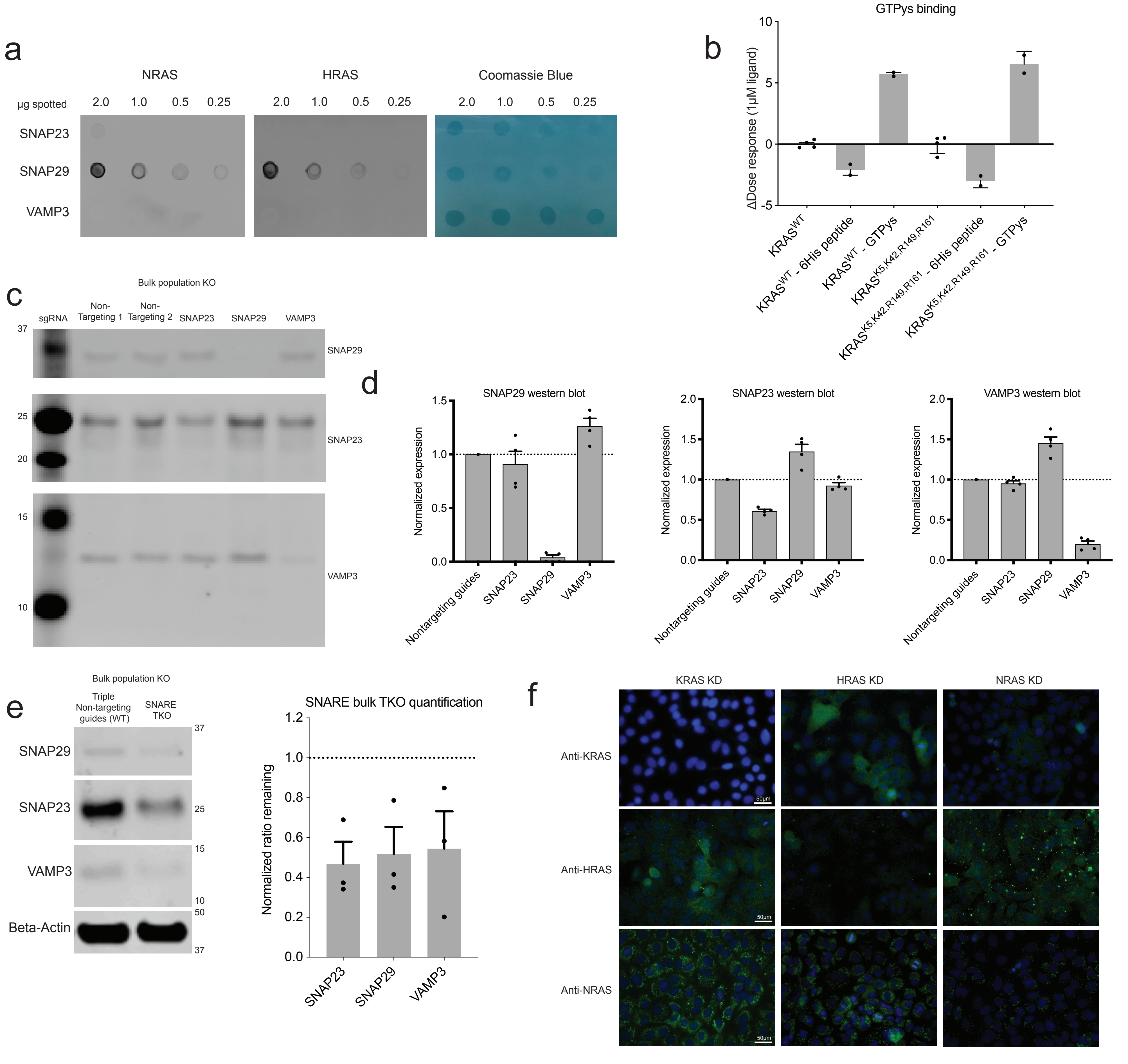

Supplementary Figure 3.
(A) Far western blot with spotted purified recombinant protein indicated by row name. Bound recombinant NRAS and HRAS were detected with an anti-RAS antibody. Coomassie stain for total protein loading (B) Dose response of KRASWT and KRASK5I, K42I, R149I, R161I and 6xHis peptide to GTPys measured by MST. (C) Representative western blots and diagram of CRISPR mediated SNARE KO in H23. (D) Quantitation of KO efficiency. (E) Representative western blots and schematic of bulk population SNARE triple KOs and quantitation from 3 biological replicates in H23. (F) Immunofluorescence of KRAS, HRAS, and NRAS in the presence of KRAS, HRAS, and NRAS KD in A549. Error bars are s.e.m.

Supplementary Figure 4

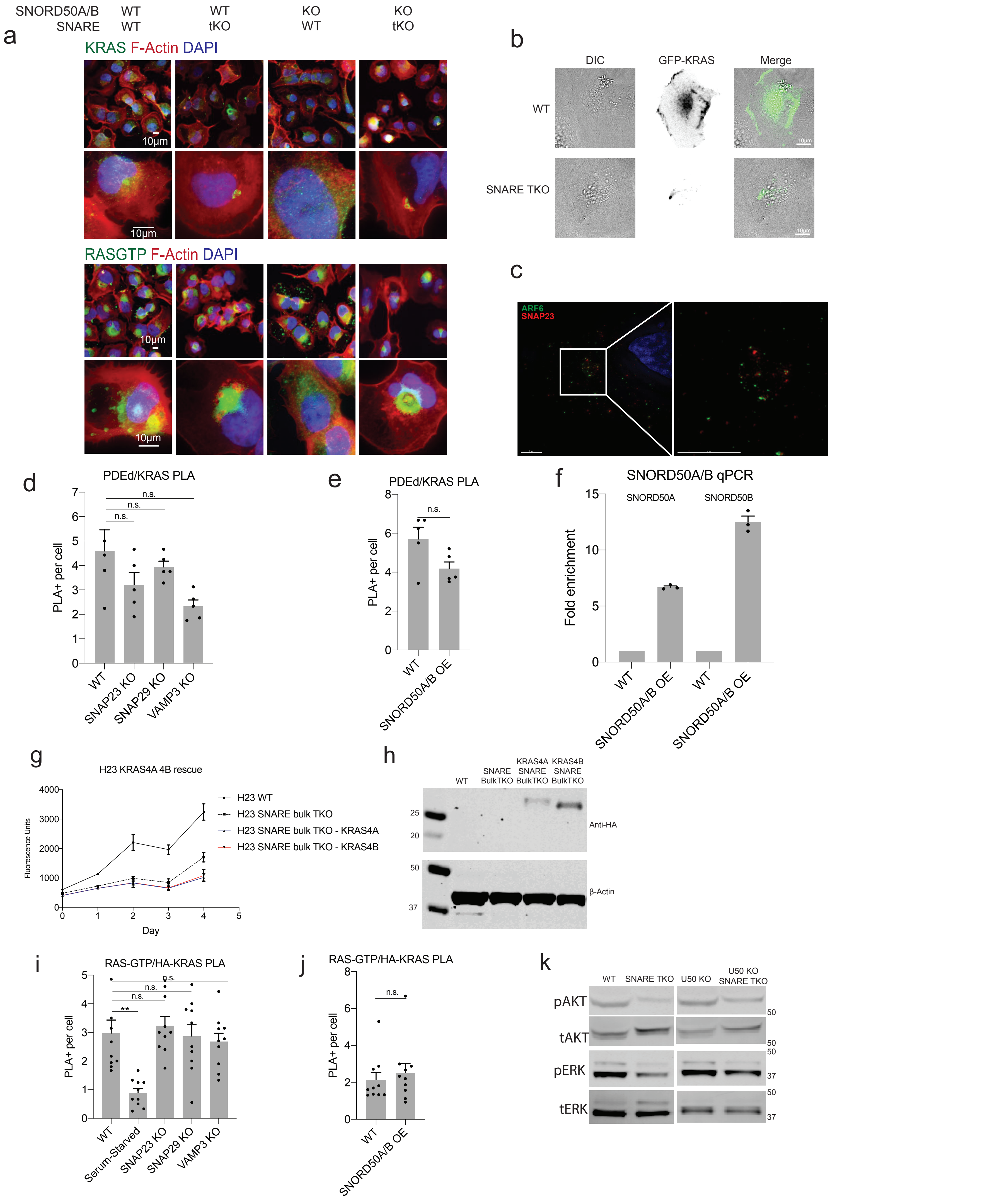

Supplementary Figure 4.

(A) Subcellular localization of KRAS as a function of SNARE bulk TKO and SNORD50A/B KO in H23. (B) Live cell imaging of A549 expressing GFP-KRAS (green) in WT and SNARE TKO conditions. (C) SNAP23 localization with ARF6 measured by super-resolution microscopy in A549. Scale bars are 5μm. (D) PLA using antibodies targeting PDEd and KRAS in SNARE KOs and in (E) SNORD50A/B overexpression (OE) (F) Verification of SNORD50A/B overexpression by qPCR. Normalized to qPCR of L32. (G) Cellular proliferation of H23 in response to SNARE bulk TKO and KRAS4A or KRAS4B rescue. (H) Immunoblot of exogenous KRAS expression in H23. (I) PLA positive interactions per cell between antibodies specific for GTP-bound Ras and HA-tagged WT KRAS in SNARE KOs and (J) SNORD50A/B overexpression. (K) Immunoblots of active/total AKT and ERK in H23. Error bars are s.e.m.

Supplementary Figure 5

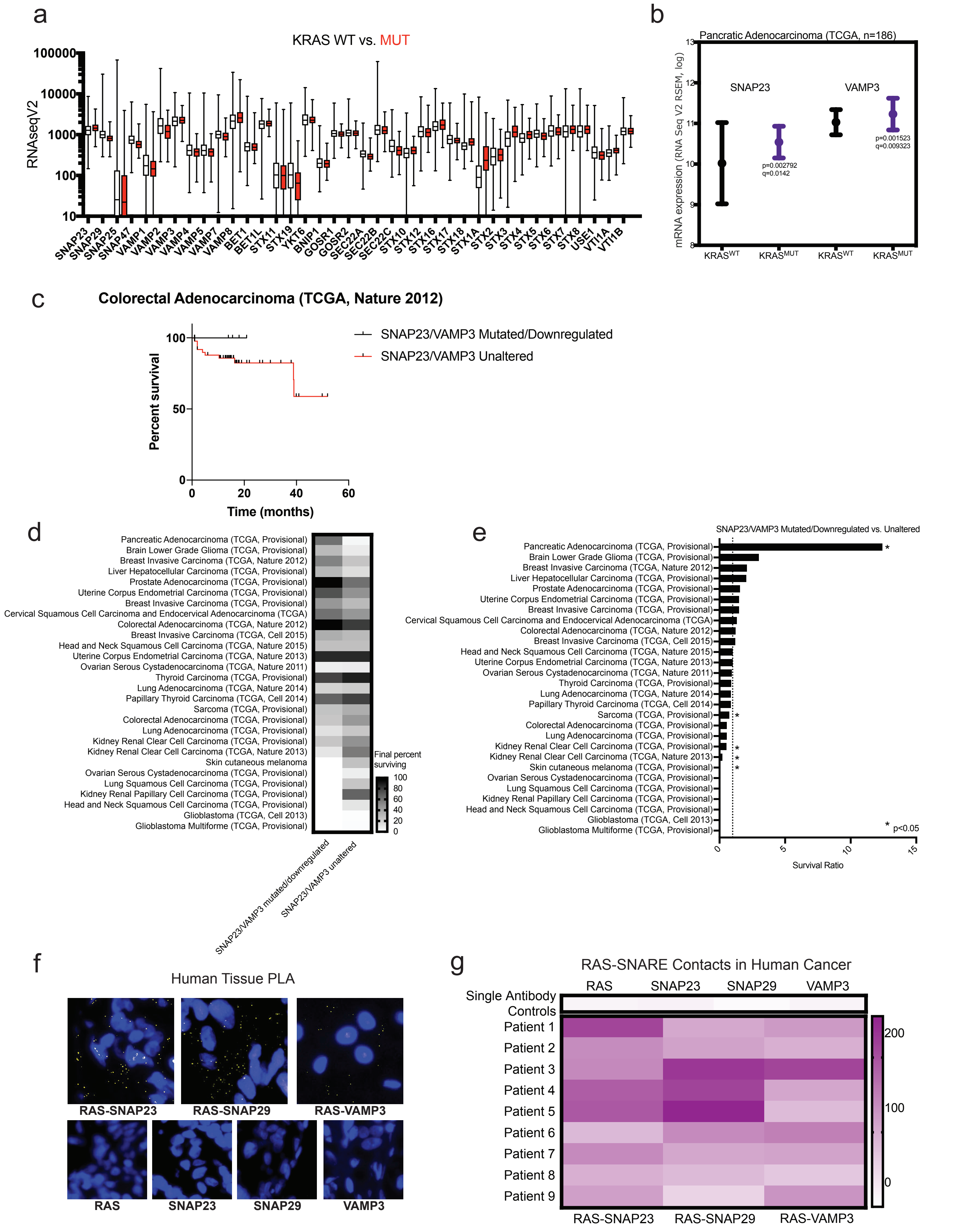

Supplementary Figure 5.
(A) SNARE expression by RNAseq in KRAS WT and MUT TCGA patients. (B) SNAP23 and VAMP3 expression by RNAseq in TCGA pancreatic adenocarcinoma patients. (C) Colorectal adenocarcinoma TCGA patient survival. (D) Percent final survival of patients with SNAP23 or VAMP3 mutation/downregulation compared to unaltered for different cancer types. (E) Ratio of final percent surviving in SNAP23/VAMP3 mutation/downregulation cohort compared to unaltered for each cancer type. (F) Proximity ligation assays in sectioned primary human colorectal cancer tissue where foci indicate proximity of Ras with the indicated SNARE proteins. (G) 9 primary colorectal cancer tissue samples were assayed as described in (F) and number of detected interactions are quantified here. Top row indicates controls where only a single antibody was used in the PLA assay. Error bars are s.e.m.

Supplementary Figure 6

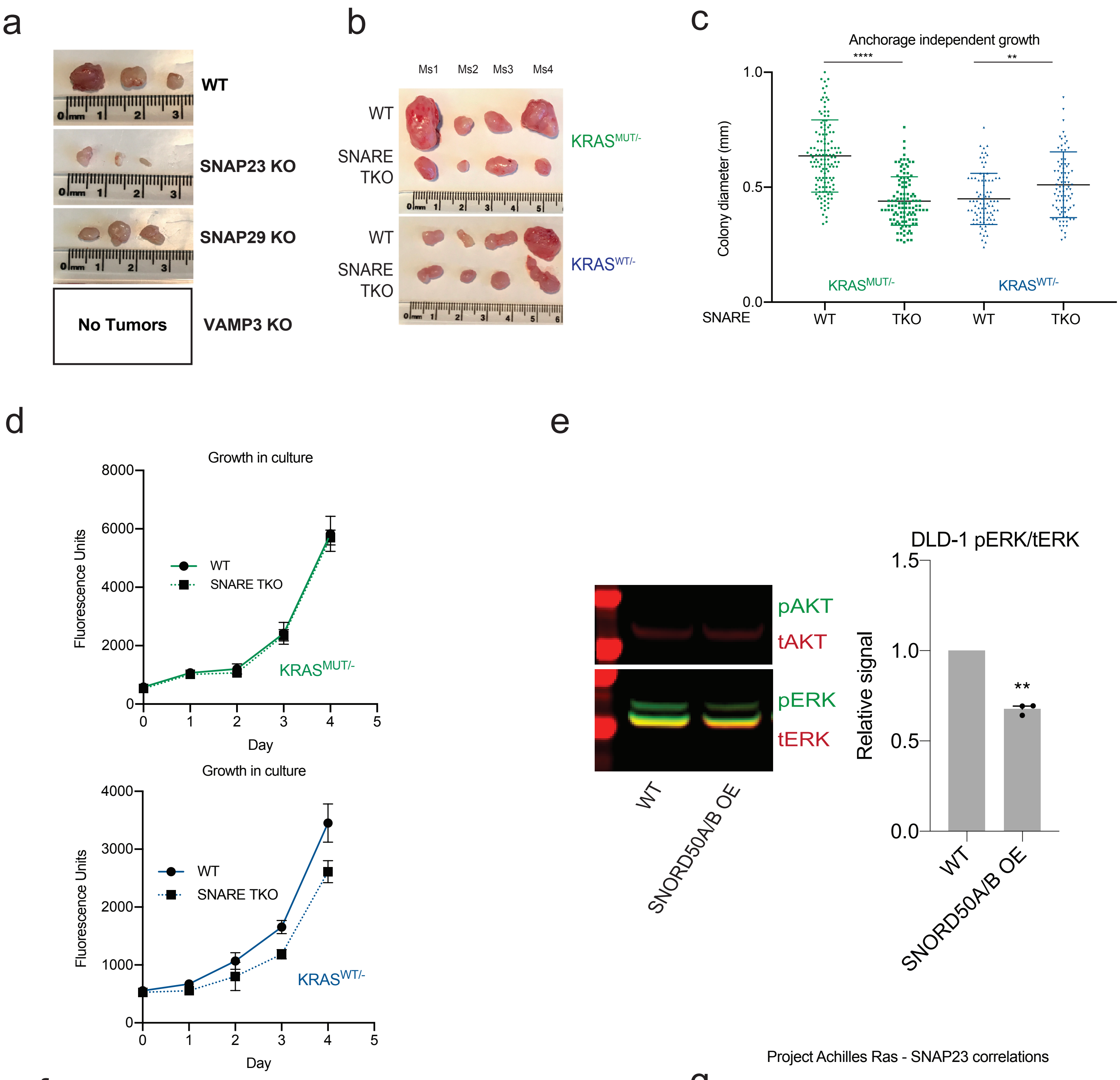

f

Empircal false discovery rate of gene-gene correlations

	KRAS	HRAS	NRAS	SNAP23	SNAP29	VAMP3	Nontargeting
KRAS	<0.001	0.013	1	<0.001	<0.001	0.621	1
HRAS	0.013	<0.001	<0.001	1	<0.001	0.574	1
NRAS	1	<0.001	<0.001	1	1	0.303	1
SNAP23	<0.001	1	1	<0.001	<0.001	0.128	1
SNAP29	<0.001	<0.001	1	<0.001	<0.001	<0.001	1
VAMP3	0.621	0.574	0.303	0.128	<0.001	<0.001	1
Nontargeting	1	1	1	1	1	1	

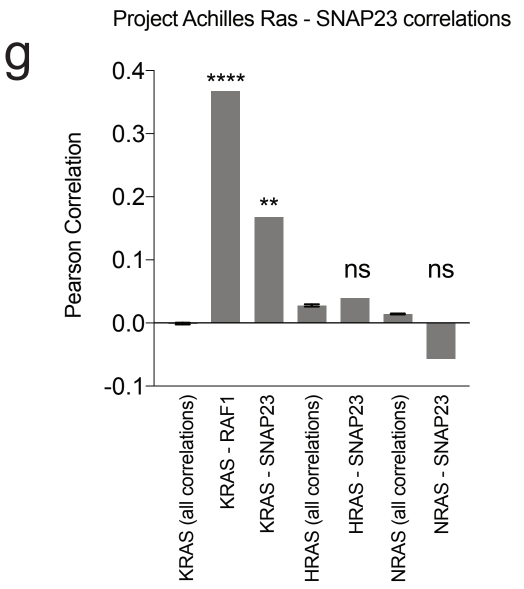

Supplementary Figure 6.
(A) Representative tumor images from H23 tumorigenesis (tumors too small to be excised were omitted). (B) Final extracted tumors from DLD-1 tumorigenesis. (C) Anchorage-independent growth and (D) growth in culture of isogenic DLD-1 cells. Error bars are mean and quartiles. (E) Overexpression of SNORD50A/B compared to mock transfection (WT) in DLD-1. Representative western blot (left) and quantitation of phosphorylated/total ERK (right, n=3) (F) Empirical false discovery rates for gene-gene correlations calculated in Fig 7b (G) Gene-gene correlations from 340 cell line whole-genome CRISPR screening from Project Achilles. Error bars apart from (c) are s.e.m.

Supplementary Figure 7

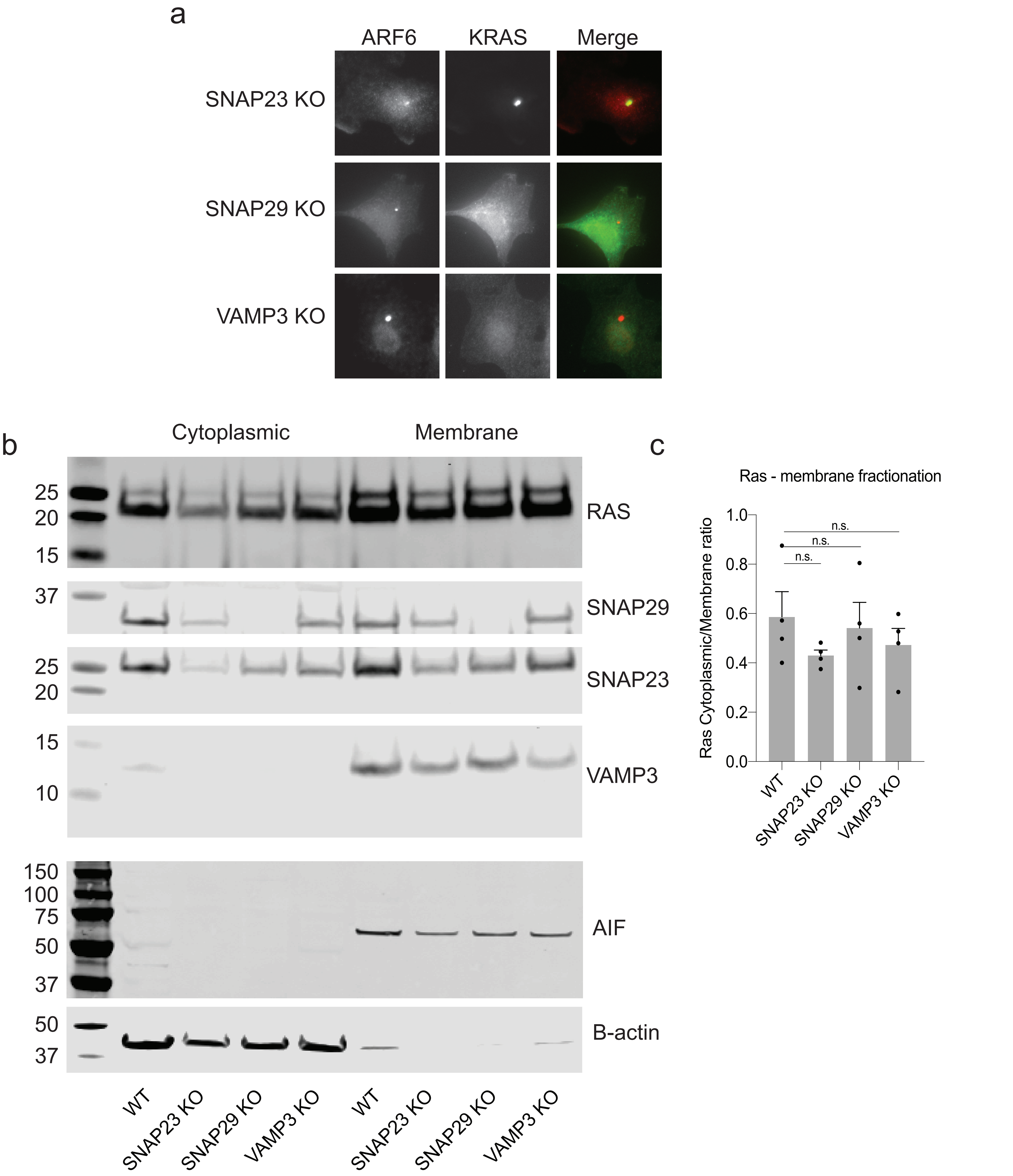

Supplementary Figure 7
(A) KRAS (green) and ARF6 (red) stained by immunofluorescence in A549. (B) Cell compartment fractionation and immunoblotting of Ras in the context of SNARE KOs. (C) Quantification of cytoplasmic/membrane ratio of Ras in (B), n=3. Error bars are s.e.m.

Supplementary Figure 8

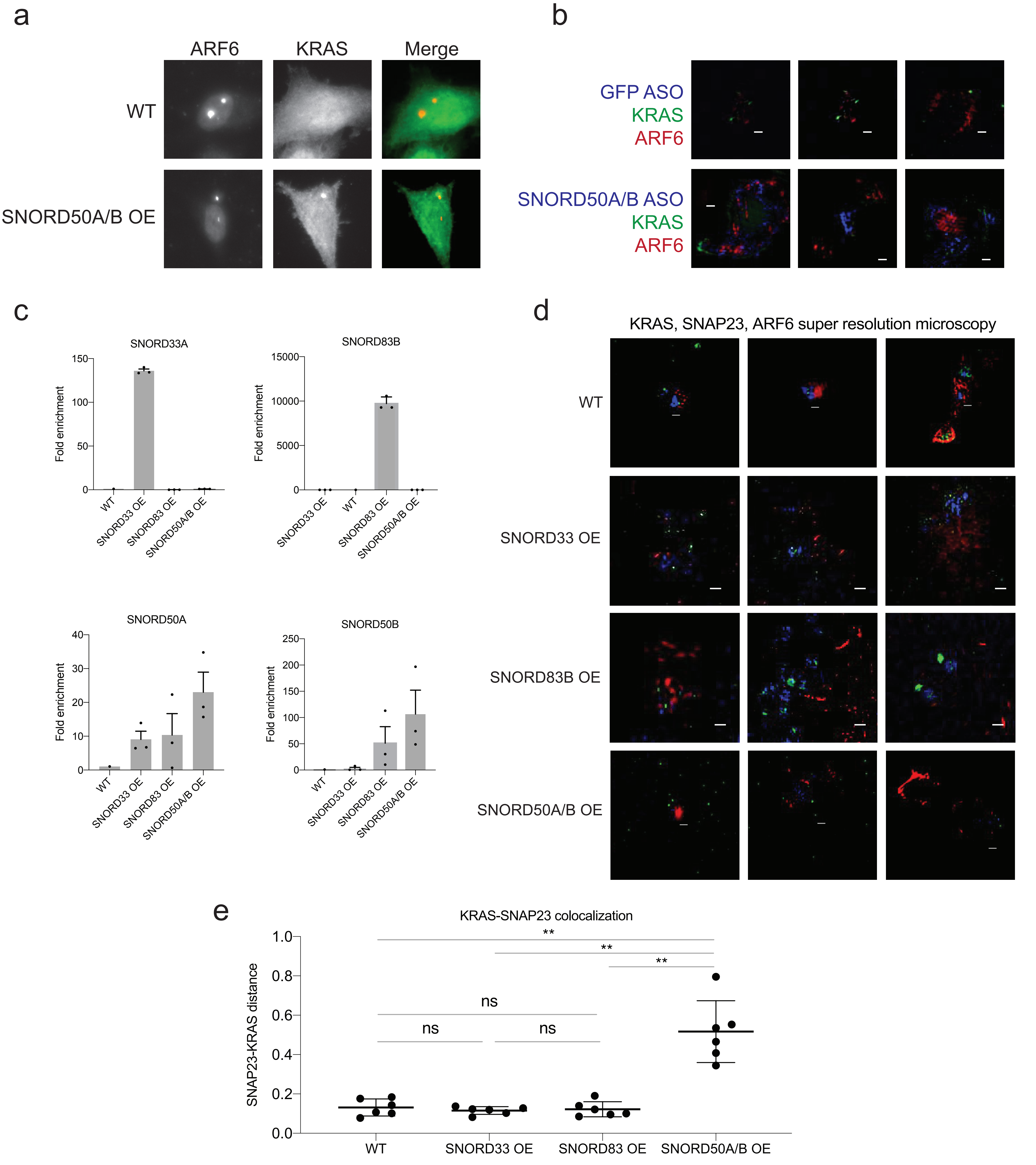

Supplementary Figure 8
(A) KRAS (green) and ARF6 (red) stained by immunofluorescence in A549. (B) Super resolution microscopy of ARF6-labeled (red) recycling endosomes with antibody co-staining of KRAS (Green) and anti-sense oligo (ASO) staining of either SNORD50A/B or GFP (Blue) in A549. (C) Quantitative PCR of snoRNA expression levels achieved in following experiments normalized to L32 controls in mock infections. Error bars are s.e.m. (D) Super-resolution microscopy of KRAS(green), ARF6 (red), and SNAP23 (blue) in A549. Three representative sections shown for each condition. Scale bar = 0.5μm. (E) Quantification of closest SNAP23-KRAS distances in ARF6-labeled recycling endosomes from (D) Error bars are mean and quartiles.

Supplementary Figure 9

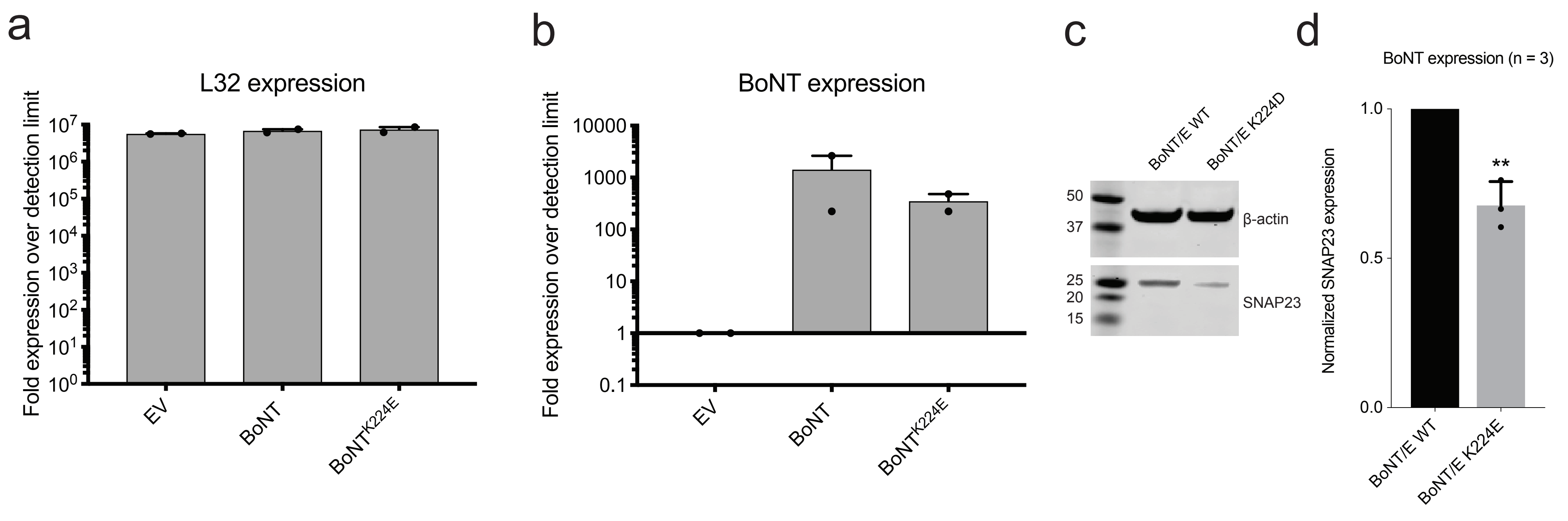

Supplementary Figure 9
(A) L32 qPCR loading control of H23 stably expressing botulism neurotoxin (BoNT). (B) qPCR of BoNT expression in the same samples as (A). (C) Western blot of SNAP23 and β-Actin in the presence of BoNT/E WT expression or BoNT/E K224D expression in the same samples from (A). (D) Quantification of (C) performed in triplicate. Error bars are s.e.m.
