# Supplementary material for: KRAS regulation by small non-coding RNAs and SNARE proteins
Source: Nat Commun. 2019 Nov 11;10:5118. doi: 10.1038/s41467-019-13106-4 (PMC6848142; doi:10.1038/s41467-019-13106-4)
Supplement: Supplementary file 3 — Description of Additional Supplementary Files [file 41467_2019_13106_MOESM3_ESM.zip]

**Description of Additional Supplementary Files**

File Name: Supplementary Data 1

Description: Mass Spectrometry Spectral Counts

File Name: Supplementary Data 2

Description: Crosslinking‐Mass spectrometry

File Name: Supplementary Data 3

Description: Human SNARE Proteins

File Name: Supplementary Data 4

Description: sgRNA Target Sequences

File Name: Supplementary Data 5

Description: RNA‐seq of SNARE TKOs (KRAS dependency signature)

File Name: Supplementary Data 6

Description: CRISPR Correlated Gene Essentiality sgRNA Counts

File Name: Supplementary Data 7

Description: TCGA Survival

File Name: Supplementary Data 8

Description: SNORD33 Protein Microarray Raw Binding Signal

File Name: Supplementary Data 9

Description: SNORD33 Protein Microarray Processed Z‐scores

File Name: Supplementary Data 10

Description: SNORD83B Protein Microarray Raw Binding Signal

File Name: Supplementary Data 11

Description: SNORD83B Protein Microarray Processed Z‐scores
