# Supplementary material for: KRAS regulation by small non-coding RNAs and SNARE proteins
Source: Nat Commun. 2019 Nov 11;10:5118. doi: 10.1038/s41467-019-13106-4 (PMC6848142; doi:10.1038/s41467-019-13106-4)
Supplement: Supplementary file 15 — Reporting Summary [file 41467_2019_13106_MOESM15_ESM.pdf]

nature research

Corresponding author(s): Paul A. Khavari

Reporting Summary

Nature Research wishes to improve the reproducibility of the work that we publish. This form provides structure for consistency and transparency in reporting. For further information on Nature Research policies, see Authors & Referees and the Editorial Policy Checklist.

Statistical parameters

When statistical analyses are reported, confirm that the following items are present in the relevant location (e.g. figure legend, table legend, main text, or Methods section).

n/a	Confirmed	
☐	☒	The exact sample size (*n*) for each experimental group/condition, given as a discrete number and unit of measurement
☐	☒	An indication of whether measurements were taken from distinct samples or whether the same sample was measured repeatedly
☐	☒	The statistical test(s) used AND whether they are one- or two-sided<br>*Only common tests should be described solely by name; describe more complex techniques in the Methods section.*
☐	☒	A description of all covariates tested
☐	☒	A description of any assumptions or corrections, such as tests of normality and adjustment for multiple comparisons
☐	☒	A full description of the statistics including central tendency (e.g. means) or other basic estimates (e.g. regression coefficient) AND variation (e.g. standard deviation) or associated estimates of uncertainty (e.g. confidence intervals)
☐	☒	For null hypothesis testing, the test statistic (e.g. *F*, *t*, *r*) with confidence intervals, effect sizes, degrees of freedom and *P* value noted<br>*Give P values as exact values whenever suitable.*
☒	☐	For Bayesian analysis, information on the choice of priors and Markov chain Monte Carlo settings
☒	☐	For hierarchical and complex designs, identification of the appropriate level for tests and full reporting of outcomes
☐	☒	Estimates of effect sizes (e.g. Cohen's *d*, Pearson's *r*), indicating how they were calculated
☐	☒	Clearly defined error bars<br>*State explicitly what error bars represent (e.g. SD, SE, CI)*

Our web collection on statistics for biologists may be useful.

Software and code

Policy information about availability of computer code

Data collection	The versions of all code used are described in full in the material and methods section. Full descriptions to recreate analyses are also provided in deposited data and materials and methods.
Data analysis	See above.

For manuscripts utilizing custom algorithms or software that are central to the research but not yet described in published literature, software must be made available to editors/reviewers upon request. We strongly encourage code deposition in a community repository (e.g. GitHub). See the Nature Research guidelines for submitting code & software for further information.

Data

Policy information about availability of data

All manuscripts must include a data availability statement. This statement should provide the following information, where applicable:

- Accession codes, unique identifiers, or web links for publicly available datasets
- A list of figures that have associated raw data
- A description of any restrictions on data availability

All data are available either in supplementary data or through accession codes linked in the manuscript supplement.

Field-specific reporting

Please select the best fit for your research. If you are not sure, read the appropriate sections before making your selection.

☒ Life sciences ☐ Behavioural & social sciences ☐ Ecological, evolutionary & environmental sciences

For a reference copy of the document with all sections, see nature.com/authors/policies/ReportingSummary-flat.pdf

Life sciences study design

All studies must disclose on these points even when the disclosure is negative.

Sample size	No trial data is in this manuscript. All sample sizes for experiments were determined prior to performing the experiment based on precedent of previous similar experiments using comparable methods.
Data exclusions	No data was excluded from analysis.
Replication	All experiments were performed in either technical or biological replicates. All replicates were used in the preparation of each data figure. No data was excluded.
Randomization	NA - no trial data.
Blinding	NA - no trial data.

Reporting for specific materials, systems and methods

Materials & experimental systems

n/a	Involved in the study	
☐	☒	Unique biological materials
☐	☒	Antibodies
☐	☒	Eukaryotic cell lines
☒	☐	Palaeontology
☐	☒	Animals and other organisms
☒	☐	Human research participants

Methods

n/a	Involved in the study	
☒	☐	ChIP-seq
☒	☐	Flow cytometry
☒	☐	MRI-based neuroimaging

Unique biological materials

Policy information about availability of materials

Obtaining unique materials	All materials are freely available to use either commercially or provided by us.

Antibodies

Antibodies used	All antibodies including source and unique identifier are provided in the material and methods of the manuscript.
Validation	Validation data for the key antibodies used are available in the supplementary data of this manuscript. Additional validation is available from the individual suppliers.

Eukaryotic cell lines

Policy information about cell lines

Cell line source(s)	Sources of all cell lines provided in the materials and methods of the manuscript.
Authentication	All lines were verified by ATCC or Horizon discovery.
Mycoplasma contamination	All cell lines tested negative for mycoplasma contamination.

Commonly misidentified lines (See ICLAC register)	NA

Animals and other organisms

Policy information about studies involving animals; ARRIVE guidelines recommended for reporting animal research

Laboratory animals	Charleston River SHO-SCID Females 6-8 weeks at initiation of experiment.
Wild animals	NA
Field-collected samples	NA
